# Extracellular vesicles and COPD: foe or friend?

**DOI:** 10.1186/s12951-023-01911-5

**Published:** 2023-05-05

**Authors:** Jiankang Wu, Yiming Ma, Yan Chen

**Affiliations:** Department of Pulmonary and Critical Care Medicine, The Second Xiangya Hospital, Central South University, 139 Middle Renmin Road, Changsha, 410011 Hunan China

**Keywords:** Extracellular vesicles, Chronic obstructive pulmonary disease, Pathogenesis, Exacerbation, Diagnosis, Treatment

## Abstract

Chronic obstructive pulmonary disease (COPD) is a chronic inflammatory airway disease characterized by progressive airflow limitation. The complex biological processes of COPD include protein hydrolysis tissue remodeling, innate immune inflammation, disturbed host-pathogen response, abnormal cellular phenotype conversion, and cellular senescence. Extracellular vesicles (EVs) (including apoptotic vesicles, microvesicles and exosomes), are released by almost all cell types and can be found in a variety of body fluids including blood, sputum and urine. EVs are key mediators in cell-cell communication and can be used by using their bioactive substances (DNA, RNA, miRNA, proteins and other metabolites) to enable cells in adjacent and distant tissues to perform a wide variety of functions, which in turn affect the physiological and pathological functions of the body. Thus, EVs is expected to play an important role in the pathogenesis of COPD, which in turn affects its acute exacerbations and may serve as a diagnostic marker for it. Furthermore, recent therapeutic approaches and advances have introduced EVs into the treatment of COPD, such as the modification of EVs into novel drug delivery vehicles. Here, we discuss the role of EVs from cells of different origins in the pathogenesis of COPD and explore their possible use as biomarkers in diagnosis, and finally describe their role in therapy and future prospects for their application.

**Figure:**
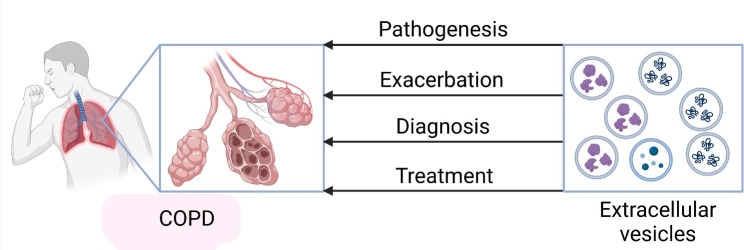
Graphical Abstract

Graphical Abstract

## Introduction

Chronic obstructive pulmonary disease (COPD) is a common, preventable and treatable chronic airway disease characterized by persistent airflow limitation and corresponding respiratory symptoms, with a pathology characterized by emphysematous destruction and remodeling of alveolar structures and narrowing and obstruction of small airways, usually associated with high exposure to harmful particles or gases [1–3]. The results of the 2019 epidemiological survey in China show that there are nearly 100 million COPD patients in China, and the prevalence of COPD in the population over 40 years of age is 13.7%, causing a heavy socioeconomic burden [4]. The development of COPD involves multiple mechanisms such as inflammation, imbalance of protein hydrolase and anti-protein hydrolase activities, oxidative stress and apoptosis, and there are interactions between different mechanisms [5]. Cigarette smoke (CS) is responsible for 80 to 90% of all COPD cases [6]. Studies have shown that the gas mixture produced by smoking contains about 4,500 components such as carbon monoxide, nicotine, oxidants and fine particulate matter. These components are thought to be strongly associated with the development of COPD [7]. CS increases the number of neutrophils, B cells, macrophages and CD8 + T cells in the small airways and lungs. These cells sequentially release a variety of inflammatory cytokines, proteases and chemokines that together lead to degeneration of the lung parenchyma [8, 9]. However, there is also growing evidence that other factors such as genetic susceptibility, in utero events and preterm birth, early life events, early or recurrent respiratory infections, and exposure to air pollution and biomass fuel fumes also play a large part in the development of COPD [10]. Symptoms of COPD include chronic cough, dyspnea and heavy sputum production, while patients with severe COPD may experience anorexia, fatigue and weight loss [11]. Cardiovascular, musculoskeletal, psychiatric disorders and diabetes mellitus are the most common co-morbidities among COPD patients, and it should not be overlooked that some chronic co-morbidities can exacerbate COPD and lead to further worsening or exacerbation of symptoms, such as depression or cardiovascular disease that can lead to decreased physical activity, weight gain and decreased lung function [12–15]. In addition, COPD patients often have acute exacerbations triggered by environmental factors including respiratory infections and air pollution [16], accompanied by increased airway and systemic inflammation, and exhibit symptoms of dyspnea and sputum exacerbation, which can severely affect the quality of life of patients [17, 18]. As acute exacerbations accelerate the long-term decline in lung function and quality of life, there is an urgent need for preventive measures and interventions to improve them. In contrast, medical management of COPD today is largely limited to temporary symptom reduction, not to control, much less individualized treatment strategies such as those for other diseases, and generally lacks meticulous, targeted therapies. Therefore, to reduce the disease burden of COPD, to clarify the pathogenesis of COPD, and to explore the diagnostic and therapeutic models for translational applications is a hot issue that needs to be addressed.

Extracellular vesicles (EVs) are a general term for nanoscale lipid bilayer vesicles released upon activation, injury or apoptosis of almost all kinds of cells; due to the complexity and heterogeneity of the biological origin of EVs, the size of vesicles is the most widely used parameter to distinguish the type of EVs. Based on particle diameter size, EVs can be classified into three categories, namely exosomes (~ 40–100 nm in diameter), microvesicles (~ 100–1000 nm in diameter) and apoptotic vesicles (~ 1000–5000 nm in diameter) [19, 20]. Apoptotic vesicles are EVs that are released by the plasma membrane as a result of programmed cell death, outward, blistering or rupture. Microvesicles are produced by the secretion of the plasma membrane outward outgrowth. Finally, as minimal exocytosis, intraluminal vesicles formed by invagination of the multivesicular endosome (MVE) membrane are released into the extracellular space after fusion of the MVE with the cell membrane [21, 22]. It has been shown that EVs have a transporting function, and EVs can mediate intercellular communication by delivering different substances (including proteins, DNA, microRNA, lncRNA, circRNA, and mRNA) to target cells [23, 24]. After being endocytosed into the cell, EVs may release their intrinsic components through three pathways, specifically by fusing with the cytoplasmic membrane, fusing with the endoplasmic reticulum, and fusing with the endoplasmic membrane or endoplasmic membrane rupture material [25]. EVs are widely distributed among the body’s tissue fluids, including blood, lymphatic fluid, cerebrospinal fluid, urine, saliva, milk, and ascites [26–29]. In addition, EVs separation can be performed by various methods, including differential ultracentrifugation, density gradient separation, and immunoaffinity capture [30]. There is growing evidence that EVs can be involved in intercellular communication in the lung and modulate pulmonary pathophysiological processes [31, 32]. There is also evidence of a relationship between exosomes and the development of chronic inflammatory respiratory diseases [33].

Due to the differences in EVs in terms of cellular origin and induced release factors, different types of EVs may both promote disease onset and progression, and may also have potential therapeutic effects on disease models, particularly respiratory diseases. In this paper, we will review the research progress of EVs involved in the pathogenesis, acute exacerbation, diagnosis and treatment of COPD in recent years, in order to provide some theoretical reference for the disease prevention and treatment of COPD.

## EVs involved in the pathogenesis of COPD

**Fig. 1:**
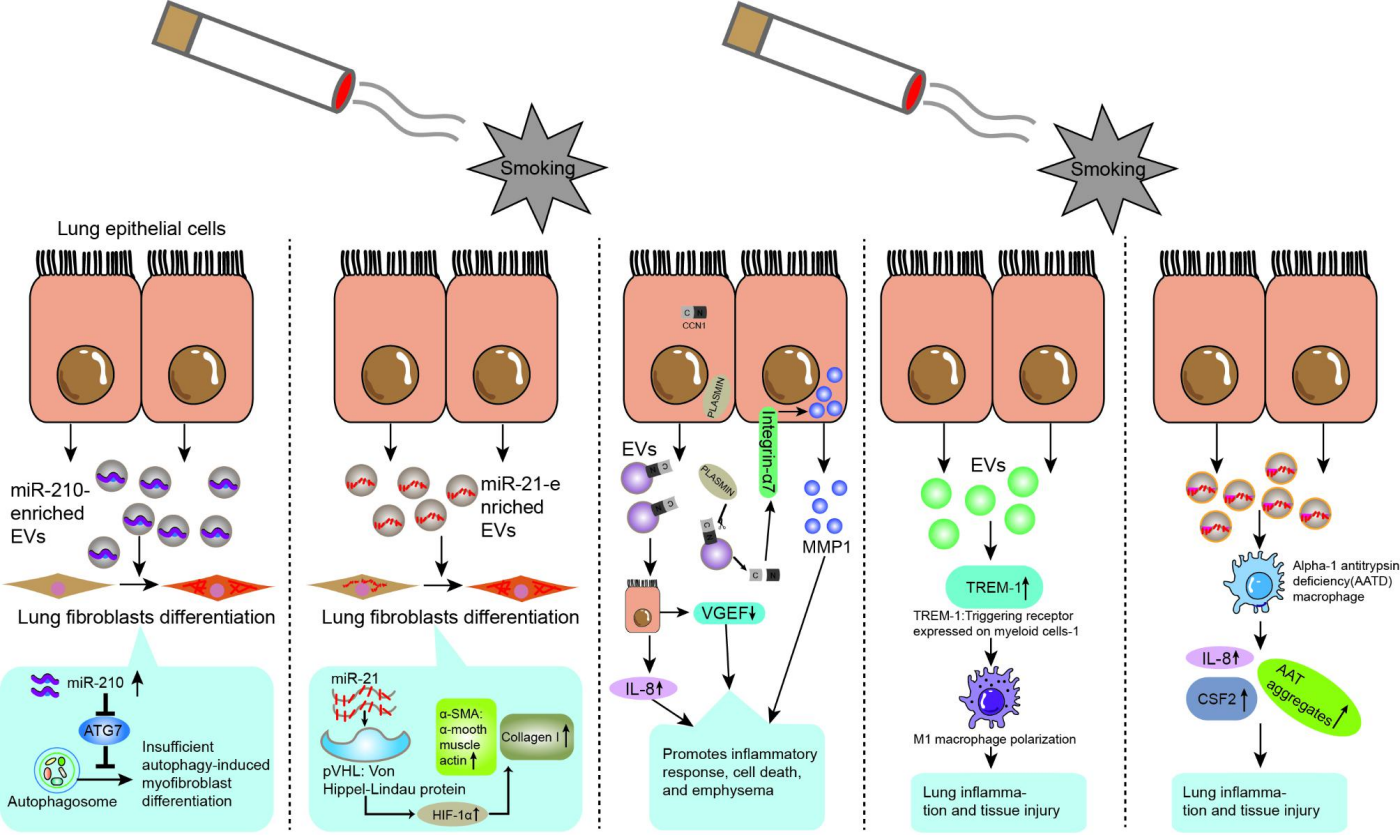
EVs of epithelial cell origin are involved in the pathogenesis of COPD

**Fig. 2:**
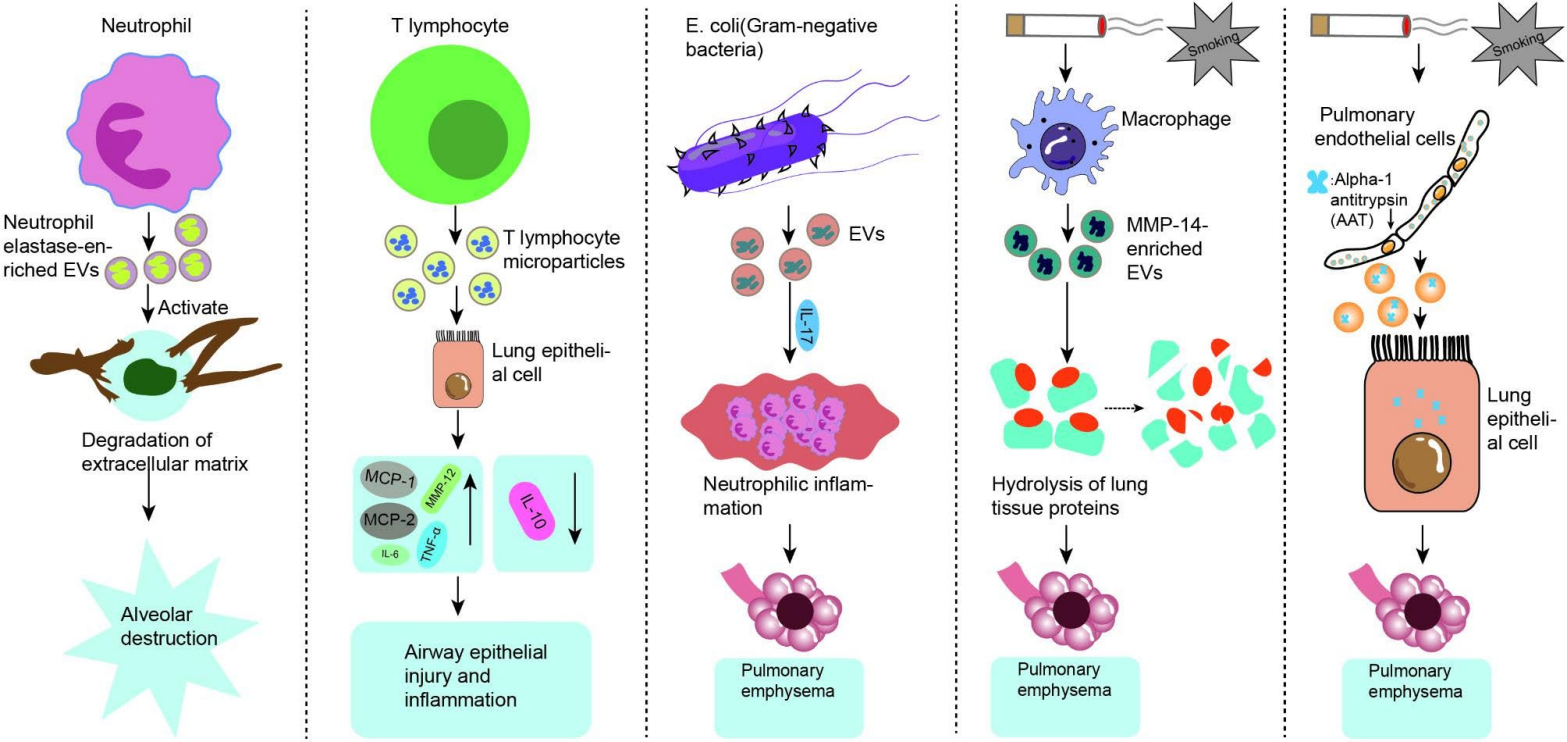
Some EVs derived from other cells are involved in the pathogenesis of COPD

A variety of cell-derived EVs are closely associated with the development of COPD, as shown in Figs. 1 and 2 and discussed in detail below.

### Epithelial cells

The three cell types that make up the airway epithelium—basal cells, cupped cells, and ciliated cells—form a basal columnar structure. As the initial line of defense for the respiratory system, airway epithelial cells prevent and remove particles and foreign objects that enter the airways with the air, so that the gas entering the alveoli is almost clean and sterile, which is essential for maintaining intrapulmonary homeostasis [34]. Additionally, it alerts nearby stromal cells and immune cells to infections and toxins that are breathed. However, the integrity and function of epithelial cells can be compromised by prolonged exposure to harmful stimuli, such as in chronic smokers [34]. Bronchial epithelial cells are main sites of contact with irritants such as CS, which induces airway epithelial damage and repair, including chemotaxis of squamous epithelial cells and proliferation of basal cells [35–37].

According to earlier research, smoking may accelerate the development of COPD by stimulating EVs from airway epithelial cell sources, as shown in Fig. 1. By transporting miR-210, Kadota et al. demonstrated that EVs of human bronchial epithelial cell origin produced in response to CS stimulation can inhibit the autophagic pathway, which in turn significantly promotes myofibroblast differentiation and is ultimately responsible for the pathogenesis of COPD [38]. One study by Xu et al. showed that human bronchial epithelium-derived exosomes stimulated by CS were able to load miR-21 to target cells, regulate the von Hippel-Lindau protein (pVHL)/hypoxia-inducible factor 1 (HIF-1) pathway, and promote epithelial mesenchymal transition, which contributed to airway remodeling in COPD [39]. Moon et al. found that after prolonged exposure to CS, full-length CCN1 (flCCN1) is secreted as an exosome and secreted fibrinolytic enzymes convert flCCN1 to cleaved CCN1 (cCCN1) in the extracellular matrix. flCCN1 promotes the production of interleukin (IL)-8 and vascular endothelial growth factor (VEGF), and IL-8-mediated neutrophil recruitment promotes cigarette smoke-induced inflammation and maintains lung homeostasis through VEGF secretion. However, cCCN1 does not function as flCCN1, which primarily promotes matrix metalloproteinase (MMP)-1 production. Both decreased VEGF and elevated MMPs contribute to the development of emphysema [40]. Recent studies have shown that CS exposure increases the inflammatory response in COPD by activating macrophages and controlling the generation of inflammatory mediators [41, 42]. In addition, CS increased the amount of EVs secreted by human bronchial epithelial cells in an in vitro COPD model, which significantly increased the polarization of M1-type macrophages by upregulating the expression of triggering receptor expressed on myeloid cells-1 (TREM-1) and ultimately exacerbated inflammatory lung injury [43]. An intriguing study also discovered that in individuals with alpha-1 antitrypsin deficiency (AATD), smoking-induced release of EVs from airway epithelial cells increased the expression of CSF2, IL-8, and alpha-1 antitrypsin(AAT) aggregates (potent neutrophil chelators) in AATD macrophages, promoting lung inflammation and damage [44].

### Endothelial cells

Pulmonary endothelial cells, also known as pulmonary microvascular endothelial cells, are structural cells of the lung, often lining the lining of microvessels where material exchange takes place. They frequently line the interior of the microvessels where material exchange occurs. They are crucial parts of the blood-gas barrier and are crucial for maintaining normal physiological and immunological function, regulating the stability of the body’s internal environment, and moderating the start, progression, and remission of disease [45]. Granules, exosomes, and apoptotic vesicles are just a few of the EVs that lung endothelial cells are capable of releasing. The small membrane-bound vesicles known as circulating endothelial microparticles (EMPs) can be released from endothelial cells in response to activation, damage, and/or apoptosis [46]. It also acts as a tool for intercellular communication [47]. EMPs are crucial for angiogenesis, endothelial function, coagulation, and inflammation. Additionally, they rise in response to numerous stimuli including endotoxins and CS[48].

Hepatocytes produce the glycoprotein serine protease inhibitor known as AATD, which is then released into the bloodstream. Although they are unable to produce AAT on their own, endothelial cells can endocytose circulating AATD to shield the lung from elastase, inflammation, and endothelial cell death [49]. Additionally, Lockett et al. demonstrated that exposure to CS drastically decreased the amount of AATD in EVs produced from endothelium, resulting in a reduction in antitrypsin transport to bronchial epithelial cells and thus favoring the development of emphysema [50].

According to some studies conducted on animals, CD42b-/CD31 + EMPs may serve as potential biomarkers for the degree of lung function impairment in rats exposed to CS [51], as well as a study finding that circulating endothelium-derived EVs increased with increasing COPD severity [52]. Although it has been proposed that endothelium-derived EVs can be used to measure the degree of COPD development, a recent clinical investigation found that, once comorbidities were taken into account, the majority of the EMPs tested were not related to the length or severity of COPD disease [53].

### Macrophages

Macrophages are essential for the body’s organs and tissues to respond innately and adaptively to outside invaders and chemicals [54]. Similar to this, lung macrophages are key players in immune surveillance, cellular debris removal, airway monitoring, and the remission of inflammation. They are typical immune cells of the pulmonary milieu [55].

Studies have shown that the amount of macrophages in the airways, lung parenchyma, bronchoalveolar lavage fluid and sputum is significantly increased in COPD patients and correlates with the severity of COPD [56]. Macrophages can release a variety of pro- and anti-inflammatory mediators, including cytokines, proteases, and protease inhibitors, which can lead to inflammation and emphysema as well as wound healing, the generation of inflammation, and the regression of inflammation [57]. Available studies have shown that in response to CS stimulation, macrophages can release matrix MMP-14 rich microvesicles that promote lung tissue proteolysis and participate in the formation of emphysema [58].

### Neutrophils

Neutrophil-derived proteases are closely associated with several chronic inflammatory lung diseases, and the disordered interaction between the anti-protease barrier and neutrophil-derived proteases is thought to be involved in the pathogenesis of several diseases, and COPD is a lung disease typically caused by a protease-anti-protease imbalance, and COPD is a typical lung disease brought on by a protease-antiprotease imbalance [59–61]. One previous study suggested the presence of activated elastase in neutrophil-derived EVs from patients with COPD, which in turn induced extracellular matrix degradation and ultimately led to the development of COPD [62]. According to a study, neutrophil-derived EVs can also cause COPD through an alpha-1 antitrypsin-resistant, neutrophil elastase(NE)-dependent mechanism in addition to elastase [63].

### T-lymphocytes

Additionally, it’s possible that the etiology of COPD involves adaptive immune responses that are similar to intrinsic immunity. Adaptive immunity mainly involves T lymphocytes, whose immune response is cellular. There are two main forms of effect of cellular immunity: direct killing of target cells after specific binding to them; the other is the release of lymphokines, which ultimately amplifies and enhances the immune effect [64]. T-lymphocyte-derived EVs from COPD patients have been demonstrated to stimulate the release of pro-inflammatory factors from bronchial epithelial cells (TNF, IL-6, MCP-1, MCP-2, and MMP-12), inhibit the synthesis of anti-inflammatory factors (IL-10), and cause airway inflammatory damage [65].

### Bacteria

Gram staining divides bacteria, the most prevalent of all organisms, into Gram-positive and Gram-positive bacteria, both of which create bacterial-derived EVs that reach the extracellular environment either constitutively or under controlled conditions [66, 67]. Among these, the cell envelope’s outer membrane, also known as outer membrane vesicles, produces EVs that are derived from Gram-negative bacteria [68, 69]. Kim et al. discovered that EVs from Gram-negative bacteria, including E. coli, can cause emphysema by encouraging neutrophil inflammation that is dependent on IL-17 A [70]. Streptococcus pneumoniae can create an endocytic deoxyribonuclease associated with EVs that aids in escaping the neutrophil extracellular traps(NETs) and thwarts the innate immune system, according to experimental research in vitro on mice [71]. Since viruses or bacteria can cause up to 80% of COPD acute exacerbations [72], bacterial and viral-derived EVs may be part of their pathogenesis. It’s important to remember that indoor dust also contains EVs from microbes, and research on animals have shown that these EVs can cause neutrophilic lung inflammation, which can result in emphysema [73]. Additional research has revealed that elevated serum anti-dust EVs IgG concentrations are a distinct risk factor for COPD [74].

Further clinical research will be required in the future to confirm the function of EVs in the etiology of COPD because current studies are primarily centered on cellular or animal models and have not been extended to genuine COPD patients.

## EVs involved in the acute exacerbation of COPD

The acute exacerbation of COPD (AECOPD), which appears as a significant worsening of dyspnea and sputum-producing symptoms and frequently results in emergency room visits and hospitalization, is a periodic worsening that characterizes the natural course of COPD [75]. The most significant driver of AECOPD is respiratory infections, which boost systemic and airway inflammation in addition to the chronic inflammation of stable COPD [76]. Although bacterial and/or viral infections are the most frequent causes [77, 78], roughly 30% of patients with AECOPD are unable to pinpoint a specific cause [79]. Exosomes may have a role in the inflammatory process of AECOPD, according to studies that have showed EVs to be correlated with plasma levels of sTNFR1, IL-6, and CRP [80].

EVs might be thought of as potential biomarkers of AECOPD since they are released from a variety of cell types, are present in all bodily fluids, and include a vast number of molecules (nucleic acids, lipids, and proteins). Studies suggest that elevated microparticles in AECOPD patients may help to understand disease progression and may initiate appropriate therapy for better clinical outcomes, particularly for platelet- and monocyte-derived microparticles [81]. Additionally, AECOPD and community-acquired pneumonia (CAP) are significant causes of illness and mortality, and accurate differential diagnosis is essential. A study distinguished CAP from AECOPD by identifying the surface protein composition of a small extracellular vesicle, even in the setting of COPD-CAP co-morbidity [82]. Circulating EMPs, a particular type of EV, are membrane vesicles with a diameter of 100 nm to mm that are released from active and apoptotic endothelial cells in the bloodstream. Increased levels of E-selectin EMPs suggest that there is lung inflammation and may help identify COPD patients who are likely to deteriorate [83]. A recent study using exosome circRNA profiling and machine learning demonstrated that exosome *hsa_circ_0005045* is upregulated by PM_2.5_ and binds to the protein cargo peroxidase 2, functionally promoting AECOPD by enticing neutrophil elastase and inducing the release of tumor necrosis factor-alpha from inflammatory cells [84].

The diagnosis of AECOPD and its severity is now based mainly on clinical symptoms, with no quantifiable and specific clinical parameters. The latest Lancet document also presents the new diagnostic criteria for AECOPD proposed by the Lancet Commission, namely the presence of cough, sputum or worsening dyspnea in patients without evidence of acute cardiac ischemia, congestive heart failure or pulmonary embolism, and at least one of the following: airflow limitation, increased respiratory or systemic inflammation, and the presence of evidence of bacterial or viral infection [85]. The criteria are primarily concerned with substantial evidence, therefore EVs can be used as evidence to assess the patient’s status and can be a helpful indicator of treatment success, but further research is required because EVs are not currently well investigated in clinically actual COPD patients.

## EVs involved in the diagnosis of COPD

**Fig. 3:**
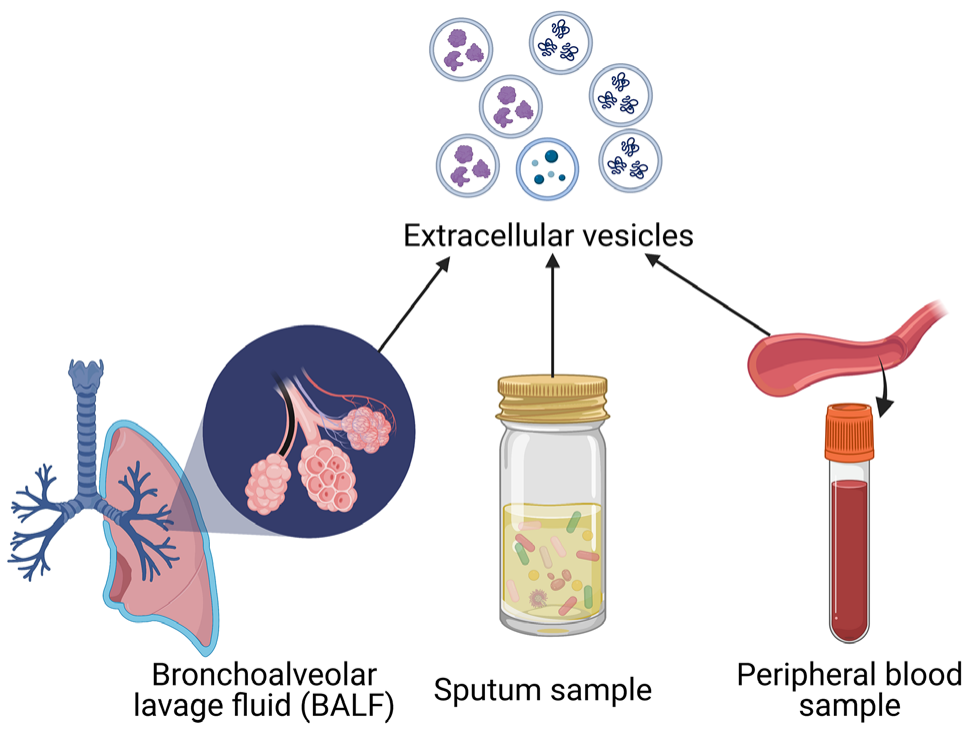
Three sources of possible EVs for diagnostic purposes. (created with BioRender.com)

The function of three separate EV sources in the diagnosis of COPD is then described below, as seen in Fig. 3.

### EVs derived from peripheral blood

Clinical diagnosis frequently involves the use of blood samples, and because serum is estimated to contain millions of EVs per microliter [86], it is vital to analyze EVs in peripheral blood. According to studies, smokers with normal spirometry but decreased lung diffusing capacity for carbon monoxide (DLCO) had elevated plasma EMP levels with apoptotic hallmarks. This suggests that plasma EMP levels can be used to monitor the early stages of emphysema [87]. The pathogenic stromal cell protein fibronectin-3 in elastic fibers demonstrated some diagnostic potential in a study using proteomic analysis of serum EVs [88]. Small non-coding RNAs (miRNAs) have important regulatory functions in a variety of cellular and biological processes, including differentiation, apoptosis, and stress resistance (immune regulation, inflammatory response, autophagy, cellular senescence, tissue remodeling, angiogenesis and tumor development) [89–91]. By separating EVs from plasma obtained from non-smokers, smokers, and COPD patients using standard isolation and identification methods, plasma-derived EV miRNAs were reported for the first time as a novel circulating lung disease biomarker in a study [92]. Exosomal miRNAs (exo-miRNAs) are the ideal candidate for circulating biomarkers since exosomes have a double membrane structure that prevents them from being degraded by ribonucleases and peripheral circulation miRNAs are vulnerable to interference from other components [93]. Three circulating exosomal miRNAs (miR-23a, miR-221, and miR-574) have been demonstrated by Shen et al. to be potential novel circulating biomarkers for the diagnosis of COPD [94]. In a recent work from Korea, a macrogenome of microbial EVs extracted from patient serum and encoded at their cumulative taxonomic levels was subjected to machine learning to create a high predictive power diagnostic model for COPD [95]. These findings imply that although circulating EVs in blood are still understudied and rarely employed in the clinical context, they are becoming more significant as prospective biomarkers for several aspects of COPD.

### EVs derived from bronchoalveolar lavage fluid (BALF)

BALF is generally a method of diagnosing and treating lung diseases by using a fiberoptic bronchoscope inserted into a segment of the bronchial lung and the fluid collected by injecting saline at 37 °C and then aspirating it with low negative pressure. In addition it is enriched with a variety of contents such as epithelial cells, macrophages, cytokines, EVs and can provide information about the different inflammatory processes occurring in the alveolar lumen [96, 97]. Studies have revealed a correlation between the frequency of AECOPD cases and microvesicles (MVs) generated [98]. COPD smokers had considerably greater levels of macrophage-derived BALF-MVs (CD14+) than did non-smokers and non-smoking COPD patients [99]. These results provide ideas for further studies on the role of BALF in COPD diagnosis in the future.

### EVs derived from sputum

Sputum, a fluid secreted by irritation of the respiratory tract, is an easily accessible, non-invasive source of biomarkers that has been widely used to assess inflammation and infection in pulmonary airway pathology [100]. Due to the inconvenience of BALF manipulation and patient intolerance, induced sputum has been suggested to replace BALF for routine clinical biomarker identification [101]. According to a study, a potential non-invasive technique to track the evolution of the disease may be the presence of CD31-MPs, CD66b-MPs, and CD235ab-MP in COPD sputum, which may reflect pulmonary endothelial damage and COPD progression [102]. Sputum has the drawbacks of being easily polluted, unstable, and vulnerable to interference, and our understanding of EVs in this medium is currently insufficient for us to consider it a valid biomarker. Therefore, more research will be required.

Although EVs have been investigated as potential biomarkers for several elements of COPD, further study is required before EVs may serve their diagnostic function in clinical practice because COPD is a complicated and heterogeneous disease.

## EVs involved in the treatment of COPD

Current advances in research on COPD suggest that EVs have the potential to be a new therapeutic direction. There are two general approaches to treatment through EVs: (1) removal of EVs containing nucleic acids or proteins involved in disease pathogenesis, including inhibition of EVs production or secretion, capture of bloodstream EVs, and preventing receptor cells from absorbing EVs; (2) use of EVs as a source of pulmonary immunomodulators [103, 104].

Mesenchymal stem cells(MSCs) play a vital role in cell therapy because of their important proliferative, differentiation and immunomodulatory abilities. Mesenchymal stem cells are pluripotent stem cells with the ability of self-renewal and multidirectional differentiation, capable of differentiating into osteoblasts, adipocytes and many other cells [105, 106]. MSCs can be obtained from umbilical cord blood, placental tissue, adipose tissue, and lung tissue in addition to bone marrow, where they are primarily found [107]. Notably, it has been shown that MSC-derived EVs could have therapeutic effects comparable to those of MSCs, are simpler to store and use, and do not cause carcinogenesis or embolization, two drawbacks of cell treatment [108]. MSC-derived EVs are EVs secreted by MSCs at rest or in response to different stimuli. In animal models of COPD intravenous, intratracheal and endobronchial injections of MSCs significantly attenuated emphysematous changes and significantly improved lung function [109]. The role of MSCs is mainly attributed to the activity of MSC-derived products (conditioned media and EVs), and EVs deliver, by paracrine and endocrine means, to immune cells, endothelial cells and alveolar epithelial cells, proteins, lipids, DNA fragments and mRNA, regulating their functions [110].

**Fig. 4:**
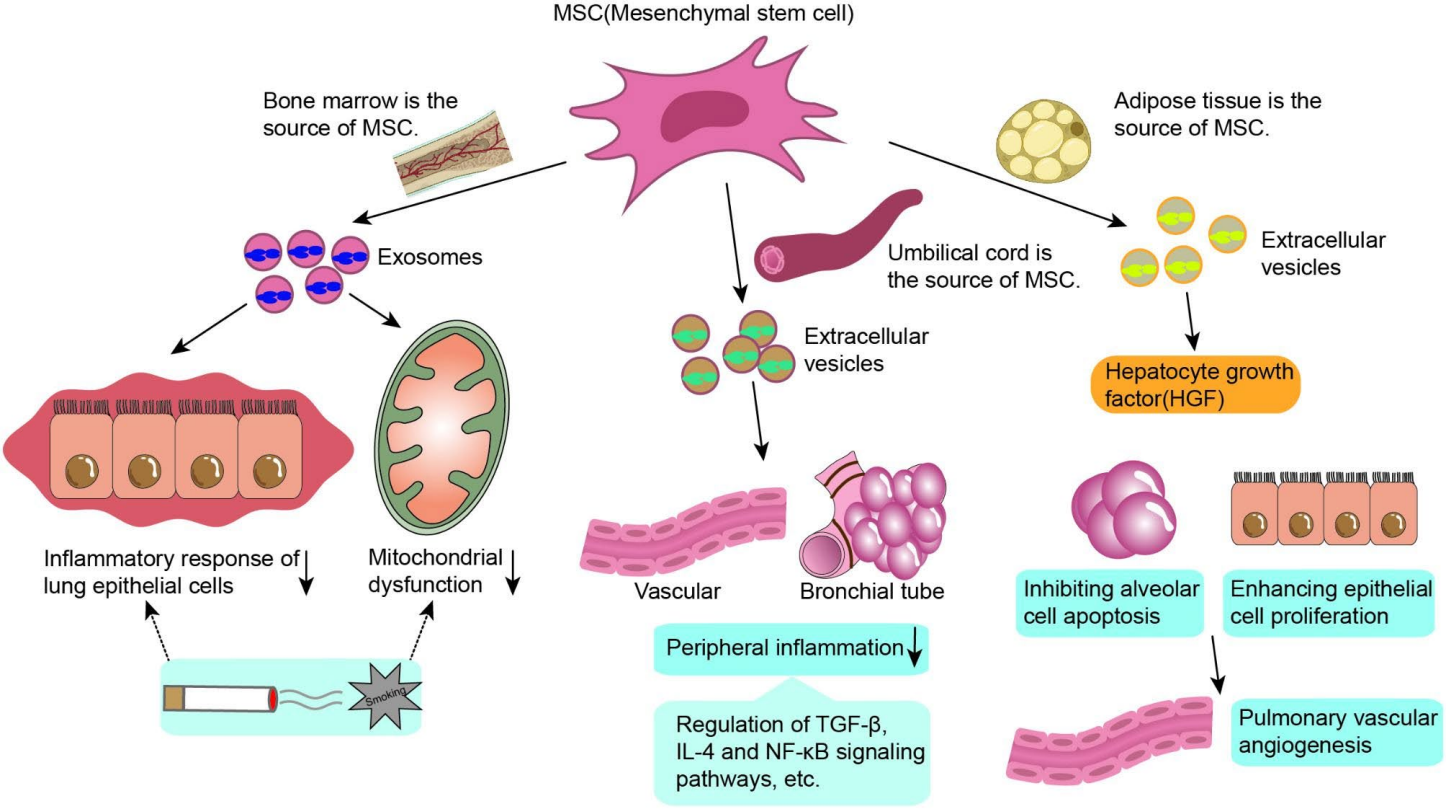
Mesenchymal stem cell-derived EVs in the treatment of COPD

Several studies have demonstrated that MSC-derived EVs, such as bone marrow, cord blood, and adipose-derived MSCs, also as seen in Fig. 4, may have a protective effect on COPD etiology. Maremanda et al. suggested that exosomes produced from mouse bone marrow mesenchymal stem cells reduced inflammation and mitochondrial dysfunction in mouse and human lung epithelial cells after exposure to CS [111]. The other study has shown that human umbilical cord MSC-derived EVs can suppress peribronchial and perivascular inflammation, and in-depth gene pathway analysis has demonstrated that EVs significantly modulate a number of known pathways associated with the pathogenesis of COPD, including the transforming growth factor-β (TGF-β) receptor signaling pathway, IL-4 signaling pathway, and NF-κB signaling pathway, among others [112]. Shigemura et al. first demonstrated that adipose tissue-derived stromal cells (ASCs) can produce hepatocyte growth factor (HGF) and promote pulmonary vascular angiogenesis by inhibiting alveolar cell apoptosis and enhancing epithelial cell proliferation, leading to restoration of lung function [113]. Notably, Kim et al. generated artificial nanovesicles from ASCs by a continuous permeation technique through polycarbonate membranes and found that in animal models of emphysema, low doses of ASC-derived artificial nanovesicles may have beneficial effects similar to high doses of ASCs or ASC-derived natural exosomes, mainly by activating the fibroblast growth factor(FGF)-2 signaling pathway to reduce the mean linear intercept (MLI) [114]. However, determining the therapeutic efficacy and optimal route of administration of EVs in the clinical setting remains the focus of future research.

**Fig. 5:**
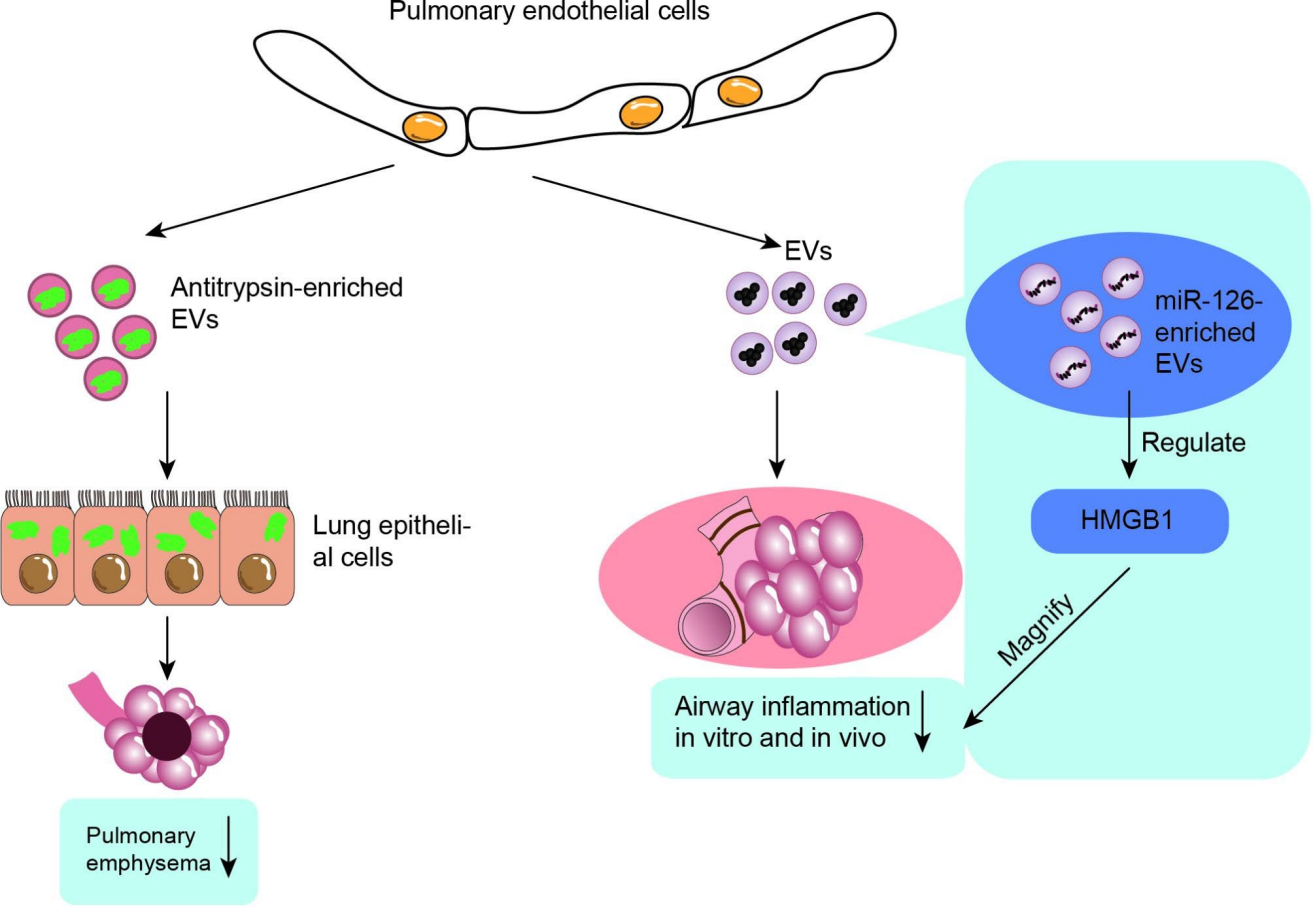
EVs of non-stem cell sources in the treatment of COPD

MSCs are currently the most popular type of cell employed in cell therapy, they are known to be relatively difficult to obtain and prone to ethical issues. Some of the most prominent ethical issues currently associated with the application of stem cell technology in medicine include: (1) the impact on stem cell providers; (2) the difficulty of regulating the source and quality of stem cells; (3) the equity of access to stem cells for patients in need [115]. Based on the above practical difficulties of stem cell application, it is of great practical importance to find other cell therapy modalities of non-stem cell origin, also as shown in Fig. 5. A recent study showed that an elastase-induced mouse emphysema model exhibited significant endothelial cell loss, and transcriptomic analysis revealed apoptotic, angiogenic and inflammatory states in mouse lung tissue; interestingly, the study found that alveolar structural destruction in emphysema was significantly reversed by intravenous injection of healthy lung endothelial cells, which certainly introduces normal lung endothelial cells to COPD treatment [116]. It has also been shown that pulmonary vascular endothelial cells have the function of promoting lung regeneration and repair through the production of matrix MMP14 and hepatocyte growth factor [117]. Previous studies have demonstrated that normal pulmonary microvascular endothelial cells can release antitrypsin-rich EVs, which further transfer antitrypsin to pulmonary epithelial cells and ultimately inhibit the development of emphysema [50]. Additionally, Ma et al. demonstrated in an in vivo and in vitro disease model of COPD that endothelial microvesicles produced from normal primary pulmonary microvascular endothelial cells reduced the airway inflammatory response; and that endothelial microvesicles specifically transporting miR-126 may further amplify the protective effect of endothelial microvesicles against COPD airway inflammation by regulating high mobility group box-1 (HMGB1) expression [118]. Indeed, the potential mechanisms underlying the distribution, pharmacokinetics and therapeutic effects of MSC-derived EVs require very extensive studies, and more extensive and comprehensive experiments are needed to launch the best protocol for isolating and preparing MSCs for clinical use. In the future, additional different cell-derived EVs should be created for the therapy of COPD.

In recent years, EVs have been increasingly used in drug delivery due to their high biocompatibility, low immunogenicity and stability [119, 120]. There are three main approaches available today to increase the yield of EVs and to better achieve the function of delivery to a specific site, if the different cell-derived EVs mentioned above are to be used in drug delivery. The first approach is genetic engineering, where plasmid vectors are used to engineer the donor cells that produce EVs [121]. Kojima et al. designed a device to enhance the packaging of RNA and assist in the secretion of intracellular EVs with different plasmids, thus greatly increasing the yield of EVs [122]. The targeting ability of EVs can also be improved by genetic engineering, and it has been shown that high affinity EVs can be constructed for both target cells by transfecting cells to express specific receptor proteins on EVs membranes [123]. The second approach is the chemical modification of EVs membranes, which allows direct coupling of ligands to the surface of EVs by chemical reactions [124]. The third approach is membrane fusion technology, which not only facilitates drug delivery, but also increases yield. In one study, by fusing vesicle membranes with functionalized liposomes, the surface of EVs could be more easily modified to facilitate binding to target cells [125]. In addition, Jhan et al. achieved a significant increase in EVs production efficiency by combining EVs with lipid-based materials [126]. Nowadays, the two main types of drug therapy for COPD are oral drugs and inhaled drugs. For inhaled drugs there are several advantages: First, inhaled drugs can directly enter the patient’s tracheobronchial tubes and have a fast onset of action. Second, inhalation drugs have a small particle diameter, which can be evenly deposited in the patient’s airways after inhalation, which is very helpful for improving the patient’s condition. Third, inhalation drugs have fewer systemic side effects compared to oral and intravenous drugs [127]. However, inhaled drugs also have some drawbacks, first of all, each inhaled drug apparatus has a different dose and method of delivering the drug to the airway [128]. In addition, the characteristics and severity of the patient’s different diseases, as well as the ease with which the patient learns the inhalation apparatus, may have an impact on the correct delivery of inhaled medication [129, 130], which in turn may affect patient compliance [131]. More importantly different devices may not be interchangeable between patients [132]. Therefore, EVs drug delivery systems for the treatment of COPD could be further investigated in the future, thus having the advantages as well as reducing the disadvantages of inhaled drugs.

In addition, some of the cutting-edge methods being employed to alter and improve EVs’ therapeutic effects are also of interest. For example, a method known as exogenous modification, including co-incubation, electroporation and ultrasound, incorporates desired substances such as RNA, proteins and other compounds into or on isolated EVs, thereby increasing the therapeutic effectiveness of EVs [133–135]. It has now been shown that incubation of EVs with immunosuppressive miR-150 can produce functionally active miRNA-EVs by association [136]. There is also a method called endogenous modification, which stimulates the cells in a certain way, thus ensuring that the desired substances are incorporated into EVs during the synthesis of EVs [137]. The most popular kind of endogenous modification involves genetically altering parental cells to cause the overexpression of a certain RNA or protein, which then raises its concentration in EVs. EVs effectively carry content to target cells while protecting miRNAs from digestion and destruction [138]. Additionally, because of their great cell specificity and ability to circumvent immune system detection, these EVs decrease the likelihood of rejection [138].

## Discussion

COPD is a common inflammatory airway disease characterized by irreversible airflow limitation and has been found to affect about 10% of the population over the age of 40, making it the third most common chronic disease worldwide. Therefore, we should strive to find more effective ways to prevent and treat COPD. Currently, the use of EVs in COPD has received a lot of attention and many scientists have revealed multiple functions of EVs in this field.

First, endothelial cell apoptosis is involved in the early development of emphysema, and exposure of endothelial cells to cigarette smoke increases the release of EVs. Other cell types also release other types of EVs when exposed to cigarette smoke, such as epithelial cells, T lymphocytes, neutrophils, and monocytes. In addition, certain bacteria are known to colonize the lower airways of COPD patients, and these pathogens also release EVs. Further research must be done to determine the role that cells other than those indicated above play in the pathophysiology of COPD. Likewise, it is crucial to study the content of EVs because they may affect receptor cells, which in turn contributes to the progression of COPD. Second, EVs are a double-edged sword in COPD, transporting substances to target cells, mediating cellular communication, and altering the activity of surrounding or distant lung structural cells as well as associated immune cells. Third, a large number of studies have demonstrated the role of EVs in acute exacerbations of COPD and their diagnostic value. EVs vary with the pathophysiological state of the disease, thus providing a possible method for the diagnosis and monitoring of COPD progression, but their role in the clinical setting needs further validation. Fourth, EVs of different cellular origins have potential therapeutic roles in COPD research. Existing studies suggest that EVs from multiple sources such as normal mesenchymal stem cells, endothelial cells and adipose may have potential therapeutic effects on COPD by inhibiting inflammatory responses, emphysema formation and lung disease.

Furthermore, quantifying and isolating different subpopulations of EVs and modifying these EVs remains a serious challenge. Some important future advances to be further investigated include (1) clarifying the specific mechanisms of EVs of multicellular origin in COPD pathogenesis; (2) elucidating the role of EVs in COPD development and healing; (3) improving EVs extraction and isolation methods; and (4) facilitating their clinical application in COPD treatment by acting as therapeutic agents or drug carriers.

## Data Availability

Not applicable.

## Abbreviations

COPD: Chronic obstructive pulmonary disease
EVs: Extracellular vesicles
CS: Cigarette smoke
MVE: Multivesicular endosome
pVHL: Von Hippel-Lindau protein
HIF-1: Hypoxia-inducible factor 1
flCCN1: Full-length CCN1
cCCN1: Cleaved CCN1
IL-8: Interleukin-8
VGEF: Vascular endothelial growth factor
MMP: Metalloproteinase
TREM-1: Triggering receptor expressed on myeloid cells-1
AAT: Alpha-1 antitrypsin
AATD: Alpha-1 antitrypsin deficiency
EMPs: Endothelial microparticles
NE: Neutrophil elastase
TNF: Tumor necrosis factor
NETs: Neutrophil extracellular traps
AECOPD: Acute exacerbation of chronic obstructive pulmonary disease
CRP: C-reactive protein
sTNFR1: Soluble tumour necrosis factor receptor-1
CAP: Community-acquired pneumonia
DLCO: Diffusing capacity for carbon monoxide
BALF: Bronchoalveolar lavage fluid
MVs: Microvesicles
MSCs: Mesenchymal stem cells
TGF-β: Transforming growth factor-β
ASCs: Adipose tissue-derived stromal cells
HGF: Hepatocyte growth factor
FGF: Fibroblast growth factor
MLI: Mean linear intercept
HMGB1: High mobility group box-1
